# Job vacancies at the European Centre for Disease Prevention and Control

**DOI:** 10.2807/1560-7917.ES.2019.24.43.1910241

**Published:** 2019-10-24

**Authors:** Eurosurveillance editorial team

**Affiliations:** 1 European Centre for Disease Prevention and Control (ECDC), Stockholm, Sweden

The European Centre for Disease Prevention and Control (ECDC) invites applications for the following positions:

 

**Seconded National Experts for several areas including *Eurosurveillance***

The application deadline expires on 2 Dec 2019.

**Principal Expert Knowledge Management**

The application deadline expires on 14 Nov 2019. 

 

For more information, please visit the ECDC website: 

https://ecdc.europa.eu/en/work-us/vacancies?f%5B0%5D=deadline_date%3A1

